# Potential Target Metabolites From Gut Microbiota Against Hepatocellular Carcinoma: A Network Pharmacology and Molecular Docking Study

**DOI:** 10.1155/2024/4286228

**Published:** 2024-10-29

**Authors:** Sehar Aslam, Muhammad Qasim, Fatima Noor, Muhammad Shahid, Usman Ali Ashfaq, Samman Munir, Helal F. Al-Harthi, Mutaib M. Mashraqi, Umair Waqas, Mohsin Khurshid

**Affiliations:** ^1^Department of Bioinformatics and Biotechnology, Government College University Faisalabad, Faisalabad, Pakistan; ^2^Institute of Molecular Biology and Biotechnology, The University of Lahore, Lahore, Pakistan; ^3^Biology Department, Turabah University College, Taif University, Taif 21995, Saudi Arabia; ^4^Department of Clinical Laboratory Sciences, College of Applied Medical Science, Najran University, Najran 61441, Saudi Arabia; ^5^College of Science and Engineering, Flinders University, Bedford Park, Adelaide, South Australia, Australia; ^6^Institute of Microbiology, Government College University Faisalabad, Faisalabad, Pakistan

**Keywords:** AKT1, anti-HCC drugs, EGFR, gut microbiota, hepatocellular carcinoma, signaling pathways, therapeutic targets

## Abstract

Hepatocellular carcinoma (HCC) is a leading cause of cancer-related deaths worldwide, posing significant challenges and economic burdens on healthcare systems. Gut microbiota metabolites have shown promise in cancer treatment, but the specific active metabolites and their key targets remain unclear. This study employed a network pharmacology-based approach to identify potent metabolites of gut microbiota and their key targets. Active metabolites produced by gut microbiota were retrieved using the database gutMGene, and targets associated with these metabolites were identified using the Swiss Target Prediction tool. HCC-related targets were obtained from the GeneCards database, and overlapping targets were selected through a Venn diagram tool. An integrated metabolites–target–pathway network was analyzed to identify active inhibitors against HCC, including *p*-cresol glucuronide, secoisolariciresinol, glycocholic acid, enterodiol, and citric acid. Molecular docking tests were performed to validate the findings and assess the binding affinity of the metabolites with their target proteins. The study identified AKT1, EGFR, ALB, and TNF genes as potential therapeutic targets against hepatic cancer. The metabolites, *p*-cresol glucuronide, secoisolariciresinol, glycocholic acid, enterodiol, and citric acid, exhibited significant binding affinity with their respective target proteins. The study also revealed multiple signaling pathways and biological processes associated with the metabolites, demonstrating their preventive effect against HCC. This research utilizes a network pharmacology-based approach to identify potent metabolites of gut microbiota and their key targets for the treatment of HCC. The findings were validated through molecular docking tests, providing a foundation for future studies on anti-HCC metabolites and their mechanisms of action. Furthermore, this study offers insights into the development of novel anti-HCC drugs utilizing gut microbiota metabolites.

## 1. Introduction

Hepatocellular carcinoma (HCC), also well-known as primary liver cancer, is a highly lethal cancer that is prevalent globally [1]. According to Cancer Research UK experts, it would be the fastest-spreading cancer by 2038–2040 [2]. HCC is a lethal disease that primarily affects people with diabetes, fatty liver disease, or hepatitis C, B, and A. Risk factors related to HCC such as excessive alcohol use, smoking, a toxic diet, and some hereditary factors [3]. Liver cancer can also be caused by abnormalities in inflammatory response, cell cycle, and metabolic networks [4]. Various therapies have been explored for the treatment of HCC, including liver transplantation, surgical resection, local ablation, sorafenib treatment, and transarterial chemoembolization [5]. Despite recent advances in treatment, most therapeutic approaches and drugs have not produced satisfactory results and have adverse effects on patients. Therefore, it is essential to explore novel natural strategies for developing drugs with minimal toxicity and maximum efficacy to increase the survival of primary liver cancer patients. Graphical abstract is shown in Supporting Figure S1.

The liver is linked with the gut through the hepatic portal circulation, and the onset of HCC is directly linked with gut microbiome dysbiosis [6]. This evidence indicates that probiotics can be used to impair imbalanced gut microbiota complexity and colonization to lower the incidence of HCC [7]. Probiotic bacteria can regulate the anti-inflammatory response and stimulate cell proliferation and differentiation by promoting the growth of valuable gut bacteria involved in the synthesis of short-chain fatty acids (FAs) [8]. Furthermore, probiotic bacteria can prevent bacterial endotoxins and protect gut epithelial functions by inhibiting the migration of gut microbes and their beneficial products into the host liver [9]. Probiotic bacteria and their metabolites also help maintain gut epithelial integrity by preventing the apoptosis of epithelial cells and stimulating the expression level of tight junction proteins [10]. Endotoxins produced by negatively altered gut microbiota are suggested as the primary risk factor for the pathogenesis of HCC through chronic hepatic inflammation [11]. Probiotic bacteria, specifically *Bifidobacteria* sp. and *Lactobacillus* sp., can reduce the incidence of HCC by preventing insulin resistance and fatty liver in obese patients [12]. Probiotics can also lower the pathogenesis of HCC via their antiviral effect against hepatitis C virus and hepatitis B virus [13, 14].

Network pharmacology (NP) is a multidisciplinary approach in the field of drug research that integrates systems biology with pharmacology [15]. This approach has shifted from the concept of one-target, one-drug to multitarget (network)—multiple therapeutic components [16]. It constructs linkages of disease–target protein–drug by using networks and available resources such as proteins, genes, diseases, and their treatments [17]. Molecular docking tests (MDTs) facilitate NP by indicating the interactions of ligands and protein receptors in in silico analysis [18]. MDTs are also useful for predicting the interactions of new drugs with protein receptors and their effects [18]. Yu et al. have demonstrated that a NP-based method could effectively find the potential metabolites of gut microbiota for treating HCC [19]. Consequently, NP is a strong technique for investigating the functions of gut microbiota and their metabolites against diseases.

The purpose of the current study was to investigate the intricate and multifaceted processes related to gut microbiota (GM), their metabolites, their targets, and signaling pathways in HCC using NP. The study aimed to identify the active metabolites and interacting genes of target proteins, utilizing complex network analysis, with the goal of enhancing treatment for HCC. To validate the potential associations and interactions between the metabolites and key protein targets, a MDT was conducted. This research holds promise for comprehending the intricate molecular mechanisms by which metabolites act against HCC and can contribute to expediting the drug discovery process.

## 2. Material and Methods

### 2.1. Active Metabolite Screening

The GM and their putative metabolites were obtained using the gutMGene database (https://bio-annotation.cn/gutmgene/) on February 4, 2023 [20]. The drug-likeness (DL) parameter and bioavailability factor were applied to virtually screen all metabolites, as these factors play a significant role in the absorption, distribution, metabolism, and excretion (ADME) properties of drugs. A tool SwissADME (https://www.swissadme.ch) was used to identify the bioavailability and DL of all metabolites on February 6, 2023 [21]. The chemical structures, molecular weights, and CID numbers of screened metabolites were obtained from PubChem. The putative targets of potential metabolites were collected from Similarity Ensemble Approach (SEA) and Swiss Target Prediction (STP) databases [22, 23]. The SEA analysis data were obtained from https://sea.bkslab.org/ on February 4, 2023 [24]. The targets related to metabolites were identified using the SMILES number format of each metabolite with the tool STP (https://www.swisstargetprediction.ch) on February 4, 2023 [25]. The metabolites meeting the criteria of probability ≥ 0.7 were selected for further analysis.

### 2.2. Screening of HCC-Related Disease Targets

Targets related to HCC were obtained from GeneCards (https://www.genecards.org) on February 5, 2023 [22, 26]. These databases give functional annotation and genomic information for known genes of humans. Duplicate genes were removed from the final gene list, and the target genes for metabolites were intersected with the HCC-related targets. Common targets were predicted using a Venn plot for further analysis.

### 2.3. Gene Ontology (GO) and KEGG Pathway Analysis of Target Metabolites

The gene annotation and gene enrichment analysis were performed using the Online Database for Annotation, Visualization, and Integrated Discovery (DAVID) (https://david.ncifcrf.gov) [27]. DAVID is a useful tool for identifying targets in three important levels: biological process, cellular component, and molecular function. In this study, the potential targets were analyzed using DAVID, and a probability cutoff of ≤ 0.05 was applied to select the top 10 KEGG pathways and top 10 GO enrichment analyses.

### 2.4. Network Construction

To explore the mechanism of target metabolites in HCC, a network analysis was performed using Cytoscape 3.8.0 software. This freely available GUI allows for the import, analysis, and visualization of biomolecules and their interactions in the generated network. The target metabolites and potential target genes were displayed as nodes, and their interactions were represented as edges. Additionally, the Network Analyzer tool was applied to identify the importance of active metabolites, potential target genes, and pathways involved in network by calculating the degree of each node. The genes having the highest degree value were identified as “active genes/targets.”

### 2.5. Protein–Protein Interaction (PPI) Networks and Molecular Docking

PPI data were obtained from the STRING database (https://string-db.org) through a search tool [28]. The database identified key interacting genes and their functional interactions with a confidence score of > 0.7, given the common genes as input. The key regulatory genes and targets were identified using CytoHubba, a plugin in the Cytoscape tool. The STRING database was also used to find the coexpression of key targets.

To validate the active targets and their interactions, a MDT was performed. The 3D structures of active target candidates were retrieved from database RCSB PDB (https://www.rcsb.org) and refined using Chimera [29, 30]. The Molecular Operating Environment (MOE) tool was then used to remove ligands, solvents, and water molecules from the key targets [31]. The site finder tool of MOE tool was used to identify the active binding sites of the target proteins, and a software PyRx was used to conduct docking of active metabolites and key targets [32].

The best docking complex results with the minimum binding energy and RMSD were chosen for further investigations. The score between metabolites and key targets was considered as the core estimation criterion for filtering potential metabolites and their targets. The docking complex with the maximum binding energy value was suggested to be the most accurate. Finally, the interactions among active metabolites and putative target proteins were visualized using Discovery Studio and Chimera X tools [33, 34].

## 3. Results

### 3.1. Screening of Active Metabolites

We retrieved a total of 208 gut microbiota metabolites from the literature and the database gutMGene. Among them, 58 active metabolites were selected depending on their oral bioavailability (OB) and DL parameters. These active metabolites include trimethylamine oxide, *D*-lactic acid, 3-(3-hydroxyphenyl) propanoic acid, 3-hydroxyphenethyl alcohol, 5-OH-equol, and many others as listed in Supporting Table S1.

**Figure 1:**
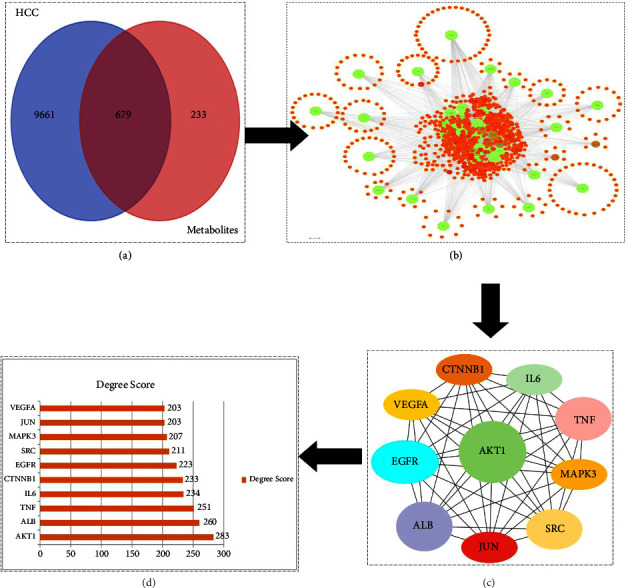
Network pharmacology-based evaluation of multitargets, multicompounds, and multiple pathways against hepatocellular carcinoma. (a) The 679 common targets between the metabolites and hepatocellular carcinoma–related targets. (b) Compounds and target network. (c) Top 10 genes ranked by degree. (d) The bar plot of the PPI network.

The STP tool was used to identify 679 common target genes of these 58 active metabolites. The GeneCards database was used to identify 10,339 genes linked with HCC. The Venn diagrams tool was implemented to figure out the mutual targets of metabolites linked to genes and HCC. As a result, we identified a total of 679 genes that could be potential key targets against HCC (Figure 1).

### 3.2. ADMET Analysis

In drug discovery, performing an ADMET analysis is a crucial process. To perform this analysis on all metabolites and represent their excellent pharmacokinetic properties, the SwissADME tool was used. The findings of the ADMET analysis indicated that the pharmacokinetic properties of all metabolites had no adverse effects. The other ADMET properties associated with metabolites for different models, including gastrointestinal absorption, blood–brain barrier (BBB) penetration, and *P*-glycoprotein substrates, revealed positive effects of 58 metabolites out of the total 208 metabolites. These results strongly suggest that these 58 metabolites could potentially act as drugs. Moreover, the Protox tool was used to identify various types of toxicity for all metabolites. The results revealed that all the active metabolites were nontoxic, and not a single metabolite showed any toxic nature.

### 3.3. Compounds–Target Network Construction

Out of the 208 metabolites from gut microbiota, 58 active metabolites were considered satisfactory. The metabolites along with their 679 target genes and their linked pathways comprise higher gene counts as all these metabolites are interlinked with multitargets, which indicates that multiple targets can produce a single synergistic response when these putative metabolites are applied as anticancer agents.

### 3.4. PPI Network

**Figure 2:**
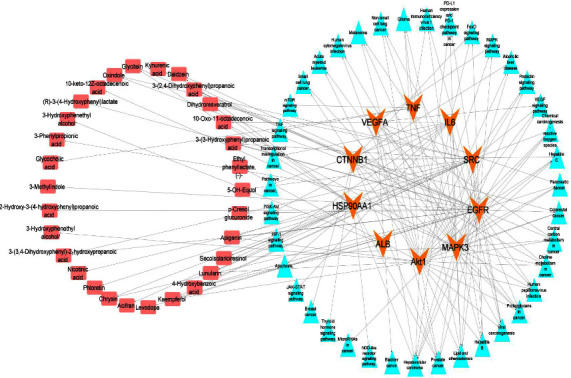
Pathways influenced by the metabolites of gut microbiota. The hub genes are depicted as orange nodes, the active metabolites as pink nodes, and the pathways associated with the key targets as sky-blue nodes.

The 679 common genes were subjected to STRING database for generating PPI network. PPI networks are useful for indicating multiple nodes, their interactions, and association with multiple targets that are involved in disease development. Later, top 10 hub genes (Akt1, EGFR, TNF, ALB, VEGFA, and SRC) were identified by submitting all common genes as an input in CytoHubba, a plug-in tool of Cytoscape (Figure 2). Afterward, the PPI network of common genes was examined through Network Analyzer, a plug-in tool Cytoscape 3.9.1. Network analyzers find the degree score of each hub gene, higher the degree score means higher association of targeted genes with other metabolites, and these target genes would be considered as active targets (Table 1). By comparing the above results with those identified through enrichment analysis, four hub genes (Akt1, EGFR, TNF, and ALB) were identified as putative antihepatic cancer targets for metabolites of gut microbiota and were further analyzed through molecular docking.

### 3.5. KEGG Pathways and GO Enrichment Analysis

**Figure 3:**
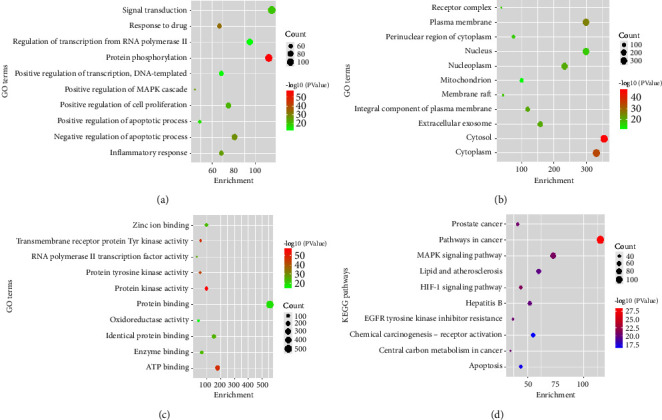
Bubble plot presentation of functional annotation and enriched pathways. (a) Gene ontology (GO) terms related to biological processes, (b) GO terms related to molecular function, (c) GO terms related to cellular components, and (d) the results of KEGG pathway analysis.

The analysis of KEGG pathways also revealed that the putative antihepatic cancer targets identified (Akt1, EGFR, TNF, and ALB) are involved in multiple pathways associated with cancer development and progression. For instance, Akt1 is involved in the PI3K-Akt signaling pathway, which plays an important role in stimulating cell survival, proliferation, and metabolism. EGFR is involved in the MAPK signaling pathway, which regulates cell growth and differentiation. TNF is involved in the NF-kappa signaling pathway, which plays a crucial role in the immune response and inflammation. ALB is involved in multiple pathways related to metabolism and cancer development (Figure 3). Overall, the functional enrichment and pathway analysis provided more insights into the potential mechanisms producing the antihepatic cancer effects of gut microbiota metabolites and highlighted the key targets and pathways that could be targeted for therapeutic interventions.

### 3.6. MDT

After performing a systematic investigation of the PPI network, we selected the four top targets: Akt1, EGFR, TNF, and ALB, for further validation of their interaction using MDTs. We obtained the 3D structures of these proteins (Akt1 (PDB ID: 3KQL), EGFR (PDB ID: 1M17), TNF (PDB ID: 2AZ5), and ALB (PDB ID: 1KTS)) from the Protein Data Bank (PDB). Using the USCF Chimera tool, we refined the structures of all four target proteins by removing all the ligands, solvents, and nonstandard residues to avoid incorrect configurations and clashes. The MDT was conducted to evaluate the potential targets of metabolites of gut microbiota that can help prevent HCC. The docking test successfully identified excellent binding association between the metabolites and the binding sites of the four target proteins. To screen for gut microbiota metabolites, we used the binding energy and docking score as key criteria. The docking complex with the highest docking score and maximum binding energy was selected. It was found that Akt1 (3KQL) had the highest binding energy and excellent conformation, as well as an RMSD value, with citric acid and glycocholic acid. EGFR (1M17) had the highest binding energy and RMSD value with enterodiol and *p*-cresol glucuronide. ALB (1KTS) had the maximum RMSD and binding energy with *p*-cresol glucuronide and citric acid. TNF (2AZ5) also showed strong bonding with *p*-cresol glucuronide and secoisolariciresinol. Therefore, our MDTs validate that the metabolites of GM bind stably with the target proteins and act as inhibitors of HCC.

The CASTp tool was used to identify the active binding sites of the targeted key proteins. The metabolites of gut microbiota were linked to the active binding site of Akt1 (3KQL) receptor by forming bonds with the amino acids TRP D28, PRO D139, GLN D25, IL3 D136, ASN D46, GLU D135, GLN D47, ALA D134, SER D133, ASN D46, and GLN D47. Similarly, for the EGFR (1M17) receptors, the metabolites bonded with the amino acids MET A742, GLU A738, LYS A721, VAL A702, LEU A820, CYS A751, MET A769, LEU A820, LEU A768, ALA A719, GLU A738, and LYS A721 residues. In addition, the TNF (2AZ5) receptors were linked to the active metabolites of gut microbiota through the following residues: GLU A198, ASP A292, ASN A279, ASP A274, SER C7, PHE A161, LEU A295, GLU A191, ASP A274, ASN A279, SER C7, LEU A295, ASP A292, and LEU A181. Furthermore, the target protein ALB showed strong interactions with amino acid residues LYS B60F, LEU B41, CYS B42, SER B195, LEU B41, GLY D193, SER B195, LYS B60F, HIS B57, TRP B60D, and GLU B192 (Table 2).

**Figure 4:**
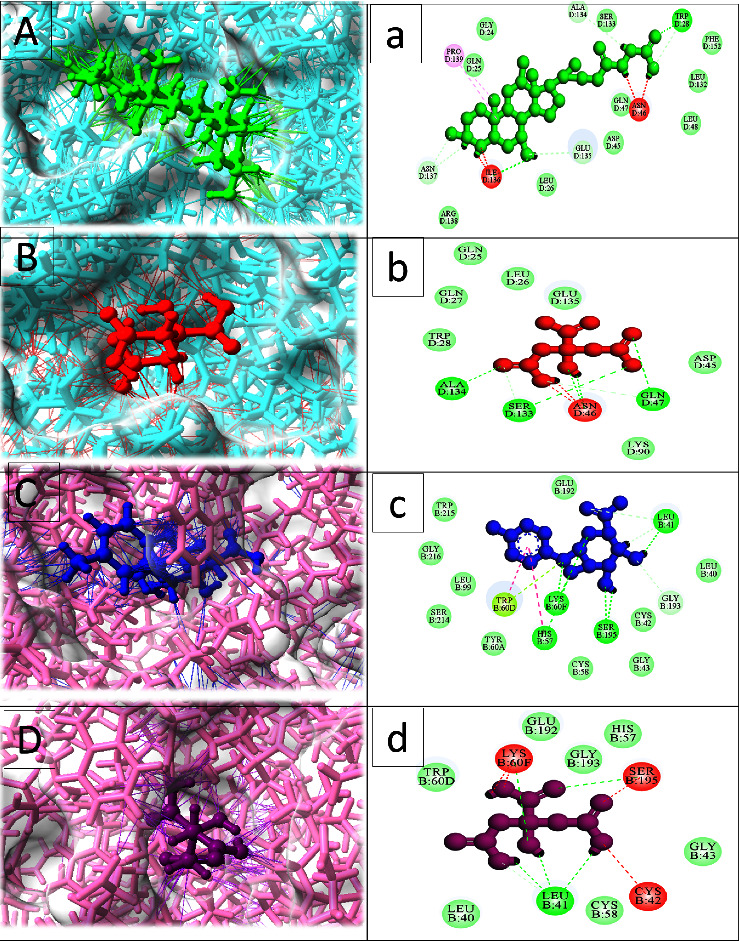
The 3D and 2D interactions between target proteins and their most potent binding metabolite components (A, a, B, b) Akt1 and (C, c, D, d) ALB.

**Figure 5:**
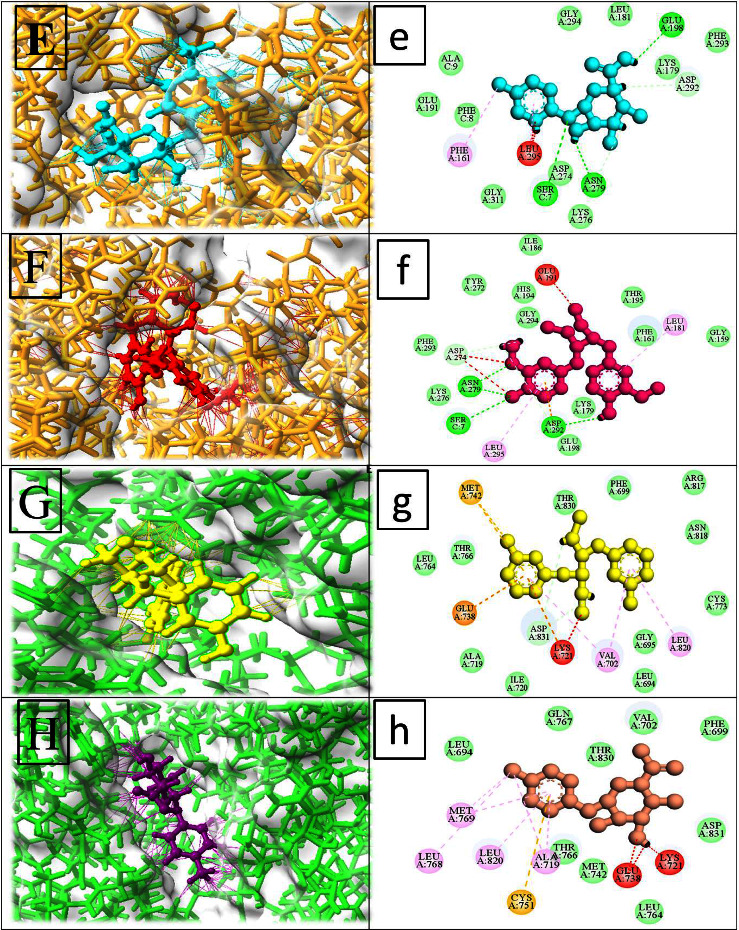
The 3D and 2D interactions between target proteins and their most potent binding metabolite components (E, e, F, f) TNF and (G, g, H, h) EGFR.

Out of 58 metabolites, the top five metabolites, namely, *p*-cresol glucuronide and secoisolariciresinol, glycocholic acid, enterodiol, and citric acid, were selected as inhibitors based on the top RMSD and highest docking score values for all target proteins (Figures 4 and 5). Notably, the molecular docking analysis revealed that *p*-cresol glucuronide interacts with EGFR, ALB, and TNF through various molecular interactions, such as hydrogen bonding, hydrophobic interactions, and *π*-*π* stacking, facilitated by its conjugated structure comprising both aromatic and glucuronide moieties. These findings suggest that these gut microbiota metabolites could be effectively used against these four target proteins for the amelioration of HCC.

Further docking studies were conducted between the control drugs and their respective target proteins. The control drugs perifosine, erlotinib, infliximab, and warfarin were evaluated for their interactions with the proteins AKT1, EGFR, TNF, and ALB, respectively. The analysis revealed that perifosine (PubChem ID: 163,092) demonstrated a binding affinity of −5.7 kcal/mol with an RMSD of 1.8 Å to AKT1, indicating a moderate binding strength and stability. Erlotinib (PubChem ID: 176,870) showed a binding affinity of −5.2 kcal/mol and an RMSD of 2.1 Å with EGFR, reflecting moderately strong and stable binding. Infliximab (PubChem ID: 25,072,267) targeted TNF with a binding affinity of −4.6 kcal/mol and an RMSD of 1.9 Å, suggesting moderate binding strength and stability. Warfarin (PubChem ID: 54,678,486) had a binding affinity of −5.9 kcal/mol and an RMSD of 1.6 Å with ALB, indicating a stable interaction and moderate binding strength.

These findings indicate that the control drugs exhibit moderate binding affinities, generally ranging from −4 to −5.9 kcal/mol, with their respective target proteins in the context of HCC. However, it is noteworthy that the metabolites identified in the current study show higher binding affinities than these control drugs. This suggests that the metabolites may have a synergistic effect, potentially enhancing their therapeutic potential by binding more strongly to these target proteins than the control drugs, thus offering promising avenues for further research and development in the treatment of HCC.

## 4. Discussion

HCC remains a significant global health challenge, and while advancements have been made in therapeutic strategies, several limitations persist. Current therapeutic modalities, including liver transplantation, surgical resection, local ablation, and systemic therapies, such as sorafenib, have shown variable efficacy and are often accompanied by significant adverse effects. Sorafenib, for example, has been a cornerstone in the treatment of advanced HCC; however, its benefits are limited by its modest improvement in overall survival, high cost, and a range of side effects including hand-foot skin reactions, diarrhea, and hypertension [35, 36].

Additionally, resistance to systemic therapies like sorafenib often develops, further complicating treatment outcomes. The genetic heterogeneity of HCC contributes to this therapeutic resistance, making it challenging to develop a one-size-fits-all treatment strategy. Moreover, the underlying liver dysfunction in HCC patients limits the use of aggressive therapies, and many patients are not eligible for curative treatments like resection or transplantation due to the advanced stage of the disease at diagnosis or other comorbidities [37].

The economic burden associated with these treatments, combined with the limited improvement in survival, underscores the need for novel therapeutic approaches that are not only more effective but also more accessible and better tolerated by patients [38]. In this context, our study aims to explore the potential of gut microbiota metabolites as a complementary therapeutic strategy that could address some of these limitations by offering a natural, multitargeted approach with potentially fewer side effects. Recently, there has been intense interest in the therapeutic values of natural products [39]. NP, a rising star in the field of drug, sheds light on concept of complex interactions between drugs, disease targets, and their possible mechanisms of action [40, 41]. However, drug development from natural sources faces challenges in their methodologies, such as the lack of ADMET properties in newly developed drugs and the high cost of research [42–44]. Thus, there is a need to develop effective drugs with better ADMET properties, minimal toxicity, and minimum cost.

The pathogenesis of HCC is also linked with the dysbiosis of gut microbiota, negative alteration of GIT epithelial barrier, and translocation of gut bacteria. Therefore, probiotic bacteria could be effectively used to overcome the risk of HCC using different mechanisms, including modulation of host gut microbiota, regulation of the immune system of host to inhibit the binding of pathogenic bacteria and viruses, prevention of gut bacteria translocation, and maintaining the integrity of gut epithelial barrier. Probiotics also stimulate the growth of beneficial bacteria that produce anti-inflammatory metabolites, which are useful in relieving hepatic-oxidative stress produced in HCC by elevating the expression level of antioxidant enzymes. Probiotic bacteria lower the risk of HCC pathogenesis by inhibiting chronic HCV and HBV infection through their antiviral activity. Furthermore, probiotics can ameliorate obesity to reduce hepatic lipotoxicity. Probiotic bacteria can downregulate the angiogenic factors ANGPTs and VEGFA through their antiangiogenic properties. More interestingly, probiotic bacteria can inhibit the expression of cancer-causing genes and upregulate the tumor suppressor genes that are involved in the pathogenesis of HCC. Probiotics can biotransform non-nutritional diet components, e.g., proanthocyanidin, into simple metabolites having antitumor activity against HCC.

The present study aimed to identify target genes involved in the pathways of different cancers, with the goal of potentially preventing the onset of disease by disrupting these cancer pathways. Previous research has shown that HCC is more common in subjects with diabetes, cirrhosis, fatty liver disorders, and hepatitis virus infections [45]. Our analysis revealed that our major targets, including AKT1, EGFR, TNF, and ALB, are significantly involved in HCC resistance pathways, which suggests that targeting these genes may disturb the pathways that lead to HCC [46]. We also found that these targeted genes are enriched in different infectious disorders such as arthritis, indicating that they can influence different anti-inflammatory cytokines and potentially affect hepatic cancer [47]. In particular, EGFR has been demonstrated to play a significant role in the regeneration of liver after chronic and acute hepatic injury, and in fibrosis and HCC [48]. Consequently, inhibiting EGFR may be useful in treating hepatic cancer. Additionally, the AKT1 gene exhibited a considerable role in tumor pathways, cell proliferation and growth, and is involved in apoptosis. Dysregulation of AKT1 has been implicated in the pathogenesis of hepatoma, further highlighting its importance as a promising therapeutic target. Overall, this study provides more insights into the potential targets for preventing and treating HCC and suggests that targeting these genes may disrupt cancer pathways and provide therapeutic benefits for patients with HCC and other related disorders [49].

The compound–target–pathway network analysis and its topological parameters identified AKT1, EGFR, ALB, and TNF as key targets for the amelioration of HCC. These potential targets were further validated using MDT which showed strong binding affinity of *p*-cresol glucuronide, secoisolariciresinol, enterodiol, glycocholic acid, and citric acid metabolites with the key targets. The multitarget binding capability of *p*-cresol glucuronide, arising from its structural versatility, positions it as a promising candidate for the development of broad-spectrum therapies targeting multiple pathways involved in HCC progression. The results of the docking test suggested that these five key metabolites can effectively target the genes AKT1, EGFR, ALB, and TNF for the amelioration of HCC.

Our study revealed significant correlations between key signaling pathways implicated in HCC and the metabolites of gut microbiota identified through our NP approach. Notably, the PI3K-Akt signaling pathway, where the AKT1 gene plays a pivotal role, emerged as a critical pathway in HCC progression. This pathway is known for regulating cell survival, proliferation, and metabolism, and its dysregulation is commonly associated with increased oncogenic potential in liver cancer [50]. The activation of AKT1 contributes to tumor growth by promoting cell survival and resistance to apoptosis, making it a promising target for therapeutic intervention.

Additionally, the MAPK signaling pathway was strongly associated with the EGFR gene, another key target in our study. The MAPK pathway is integral to the regulation of cell growth and differentiation [51]. In HCC, the overexpression or mutation of EGFR is linked to poor clinical outcomes, as it drives uncontrolled cell proliferation and contributes to resistance against conventional treatments [52]. Our findings suggest that targeting EGFR within the MAPK pathway could offer new avenues for therapeutic development.

The study also highlighted the role of the NF-kappa B signaling pathway, particularly through the TNF gene, in the pathogenesis of HCC. Chronic activation of this pathway creates a proinflammatory environment that not only supports tumor growth but also exacerbates disease progression [53]. Given its involvement in immune response and inflammation, targeting the NF-kappa B pathway could be beneficial in reducing the inflammatory milieu that fuels HCC development. These findings underscore the potential of gut microbiota metabolites as modulators of these key signaling pathways, offering promising opportunities for the development of novel therapeutic strategies against HCC.

The current clinical treatments for HCC primarily involve targeted therapies such as sorafenib, lenvatinib, and regorafenib. These therapies focus on inhibiting key pathways like VEGF, RAF/MEK/ERK, and PDGFR, which are crucial for tumor growth and angiogenesis. These drugs were developed based on the traditional single-target drug discovery approach, which, while effective in certain cases, may not fully address the complexity of HCC as it is not a single-gene disorder but involves intricate interactions across multiple biological pathways.

In contrast, our study employs a multitarget drug discovery approach, identifying naturally occurring metabolites derived from gut microbiota—specifically *p*-cresol glucuronide, secoisolariciresinol, glycocholic acid, enterodiol, and citric acid—as potential therapeutic agents. These metabolites were selected for their ability to interact with multiple key proteins (AKT1, EGFR, TNF, and ALB) involved in HCC pathogenesis, as validated through molecular docking studies. The multitarget nature of these metabolites suggests that they may exert synergistic effects, potentially leading to more effective treatment outcomes by simultaneously modulating several pathways critical to HCC progression.

Furthermore, these gut microbiota-derived metabolites offer a novel therapeutic strategy by targeting the gut–liver axis, which is not addressed by current therapies. This approach not only aims to treat the tumor directly but also to restore gut microbiota balance, thereby potentially reducing the risk of HCC progression through holistic modulation of host metabolism and immune response. Importantly, the natural origin of these metabolites suggests a lower risk of adverse effects compared to synthetic drugs, making them promising candidates for further development as complementary or alternative therapies for HCC. To sum up, NP provides a multitarget approach, which is particularly beneficial for addressing complex diseases like HCC. It allows for the identification of multiple therapeutic targets and pathways, offering a more comprehensive understanding of disease mechanisms. Additionally, this method is efficient, cost-effective, and helps in predicting potential off-target effects early in the drug development process, thereby reducing side effects. However, NP also has its limitations. It relies heavily on the quality and availability of existing biological data, which can affect the accuracy of predictions. The complexity of data integration poses challenges, and extensive in vitro and in vivo validation is required to confirm the findings. Furthermore, the approach is limited by the current state of biological knowledge, which can lead to overfitting in some cases. Despite these challenges, NP offers a promising framework for drug discovery and the development of novel therapeutic agents, providing a valuable complement to traditional methods.

The present study is the first one to use a comprehensive NP-based approach to explore the key metabolites of gut microbiota against HCC. The identified active metabolites, their respective targets, and associated pathways provide a foundation for future investigations in the field of drug discovery. This study sheds light on molecular mechanisms of the anticancer activity of gut microbiota metabolites and may inspire further exploration of novel metabolites from gut microbiota. However, to validate the potential pharmacological applications of gut microbiota metabolites, more in vitro and in vivo research work is required, which will accelerate the process of drug development.

## 5. Conclusion

Current research has established a scientific foundation for investigating the therapeutic potential of multicomponents and identified multiple drug targets, including novel targets, for curing HCC. By employing a NP and MDT-based approach, this research uncovered the underlying molecular mechanisms for the amelioration of this cancer. The network analysis revealed that the gut microbiota contains multiple active metabolites that have potential therapeutic value against various disease-related pathways, making them promising candidates for the treatment of hepatic cancer. Additionally, the study identified AKT1, EGFR, ALB, and TNF genes as potential targets for drug intervention, and *p*-cresol glucuronide, secoisolariciresinol, glycocholic acid, enterodiol, and citric acid as active inhibitors that could be effective in mitigating the risk factors associated with HCC. However, there are certain limitations to the current study, and it is crucial to investigate and validate these findings before utilizing gut microbiota metabolites in pharmacological applications. Overall, these findings provide valuable insights into the potential therapeutic targets and metabolites for HCC and could pave the way for future investigations in this field.

## Figures and Tables

**Table 1:** Top 10 hub genes ranked by degree score.

Protein	Metabolites	Degree	Pathways
AKT1	3-Hydroxyphenethyl alcohol/lunularin/apigenin/kaempferol/ethyl phenyllactate (−)−	283	Breast cancer, hepatocellular carcinoma, colorectal cancer, MAPK signaling pathway, HIF-1 signaling pathway, JAK-STAT signaling pathway, apoptosis, viral carcinogenesis, chemical carcinogenesis–receptor activation, prolactin signaling pathway, nonalcoholic fatty liver disease, lipid and atherosclerosis, hepatitis B, hepatitis C
EGFR	p-Cresol glucuronide/oxindole/apigenin/kaempferol/chrysin/daidzein/glycitein/3-phenylpropionic acid/4-hydroxybenzoic acid/3-methylindole/2-hydroxy-3-(4-hydroxyphenyl) propanoic acid/levodopa	223	MicroRNAs in cancer, chemical carcinogenesis–receptor activation, breast cancer, chemical carcinogenesis–reactive oxygen species, Cushing syndrome, adherens junction, pathways in cancer, proteoglycans in cancer, pancreatic cancer, non–small-cell lung cancer, choline metabolism in cancer, PI3K-Akt signaling pathway, colorectal cancer, JAK-STAT signaling pathway, nonalcoholic fatty liver disease, hepatocellular carcinoma, human papillomavirus infection, HIF-1 signaling pathway, MAPK signaling pathway, prostate cancer, central carbon metabolism in cancer, hepatitis C
TNF	Glycocholic acid	251	MAPK signaling pathway, hepatitis B, lipid and atherosclerosis, hepatocellular carcinoma, apoptosis, T-cell receptor signaling pathway, human immunodeficiency virus 1 infection, glioma, human T-cell leukemia virus 1 infection, necroptosis, natural killer cell mediated cytotoxicity
IL6	10-Oxo-11-Octadecenoic acid	234	Transcriptional misregulation in cancer, TNF signaling pathway, alcoholic liver disease, MAPK signaling pathway, hepatitis B, lipid and atherosclerosis, hepatocellular carcinoma, apoptosis, T-cell receptor signaling pathway, human immunodeficiency virus 1 infection, glioma, human T-cell leukemia virus 1 infection, necroptosis, natural killer cell–mediated cytotoxicity
CTNNB1	Kynurenic acid/3-(2,4-dihydroxyphenyl)propanoic acid	233	Thyroid hormone signaling pathway, hepatitis C, colorectal cancer, Rap1 signaling pathway, breast cancer, prostate cancer, Kaposi sarcoma–associated herpesvirus infection, proteoglycans in cancer, alcoholic liver disease, C-type lectin receptor signaling pathway, choline metabolism in cancer
HSP90AA1	Lunularin/daidzein/glycitein/phloretin/acifran/secoisolariciresinol	213	PI3K-Akt signaling pathway, chemical carcinogenesis–receptor activation, lipid and atherosclerosis, Th17 cell differentiation, hepatocellular carcinoma, NOD-like receptor signaling pathway prostate cancer
SRC	Lunularin/dihydroresveratrol/oxindole/apigenin/kaempferol/chrysin/5-oh-equol/3-(3-hydroxyphenyl) propanoic acid/4-hydroxybenzoic acid/secoisolariciresinol	211	Chemical carcinogenesis–receptor activation, lipid and atherosclerosis, hepatocellular carcinoma, proteoglycans in cancer, VEGF signaling pathway, prolactin signaling pathway, thyroid hormone signaling pathway, endocrine resistance, ErbB signaling pathway, chemical carcinogenesis–reactive oxygen species, C-type lectin receptor signaling pathway, chemokine signaling pathway, viral carcinogenesis, hepatitis B
MAPK3	10-Oxo-11-Octadecenoic acid/10-keto-12Z-octadecenoic acid/secoisolariciresinol	207	MicroRNAs in cancer, chemical carcinogenesis–reactive oxygen species, breast cancer, choline metabolism in cancer, adherens junction, PI3K-Akt signaling pathway, hepatitis C, colorectal cancer, proteoglycans in cancer, non–small-cell lung cancer, human papillomavirus infection, JAK-STAT signaling pathway, HIF-1 signaling pathway, MAPK signaling pathway, prostate cancer, nonalcoholic fatty liver disease, central carbon metabolism in cancer, chemical carcinogenesis–receptor activation, Cushing syndrome, pancreatic cancer, hepatocellular carcinoma
VEGFA	Chrysin/kaempferol	203	MAPK signaling pathway, hepatitis B, lipid and atherosclerosis, hepatocellular carcinoma, apoptosis, T-cell receptor signaling pathway, human immunodeficiency virus 1 infection, glioma, human T-cell leukemia virus 1 infection, necroptosis, natural killer cell–mediated cytotoxicity
JUN	3-Hydroxyphenethyl alcohol/lunularin/apigenin/kaempferol/ethyl phenyllactate, (−)−	203	Chemical carcinogenesis–receptor activation, breast cancer, prolactin signaling pathway, pathways in cancer, HIF-1 signaling pathway, MAPK signaling pathway, apoptosis, viral carcinogenesis, hepatitis C, colorectal cancer, JAK-STAT signaling pathway, nonalcoholic fatty liver disease, hepatitis B, lipid and atherosclerosis, hepatocellular carcinoma

**Table 2:** Binding score and interactions of active metabolites along with their respective target protein.

Metabolite name	Metabolite ID	RMSD value	Docking score	Interactions
*Akt1 (2AZ5)*				
Glycocholic acid	10,140	3.379569	−12.54636	TRP D28, PRO D139, GLN D25, IL3 D136, ASN D46, GLU D135, GLN D47, ALA D134
Citric acid	311	1.8	−12.418093	ALA D134, SER D133, ASN D46, GLN D47

*EGFR (1M17)*				
Enterodiol	115,089	1.618637	12.02515	MET A742, GLU A738, LYS A721, VAL A702, LEU A820
*p*-Cresol glucuronide	154,035	0.6	−12.337878	CYS A751, MET A769, LEU A820, LEU A768, ALA A719, GLU A738, LYS A721

*ALB (1KTS)*				
*p*-Cresol glucuronide	154,035	2.402233	−13.2254	LEU B41, GLY D193, SER B195, LYS B60F, HIS B57, TRP B60D, GLU B192
Citric acid	311	2.6	−14.3668	LYS B60F, LEU B41, CYS B42, SER B195

*TNF (3KQL)*				
*p*-Cresol glucuronide	154,035	1	−14.026333	GLU A198, ASP A292, ASN A279, ASP A274, SER C7, PHE A161, LEU A295
Secoisolariciresinol	65,373	2.182394	−14.022463	GLU A191, ASP A274, ASN A279, SER C7, LEU A295, ASP A292, LEU A181

## Data Availability

The data that support the findings of this study are available in the supporting information of this article.

## Supporting Information

Figure S1: Graphical abstract illustrating the workflow used in the current study. Table S1: The selected metabolites of gut microbiota and their physiochemical properties.
