# Supplementary material for: Potential Target Metabolites From Gut Microbiota Against Hepatocellular Carcinoma: A Network Pharmacology and Molecular Docking Study
Source: Int J Microbiol. 2024 Oct 29;2024:4286228. doi: 10.1155/2024/4286228 (PMC11537736; doi:10.1155/2024/4286228)
Supplement: Supporting Information — Additional supporting information can be found online in the Supporting Information section. [file 4286228.f1.docx]

**
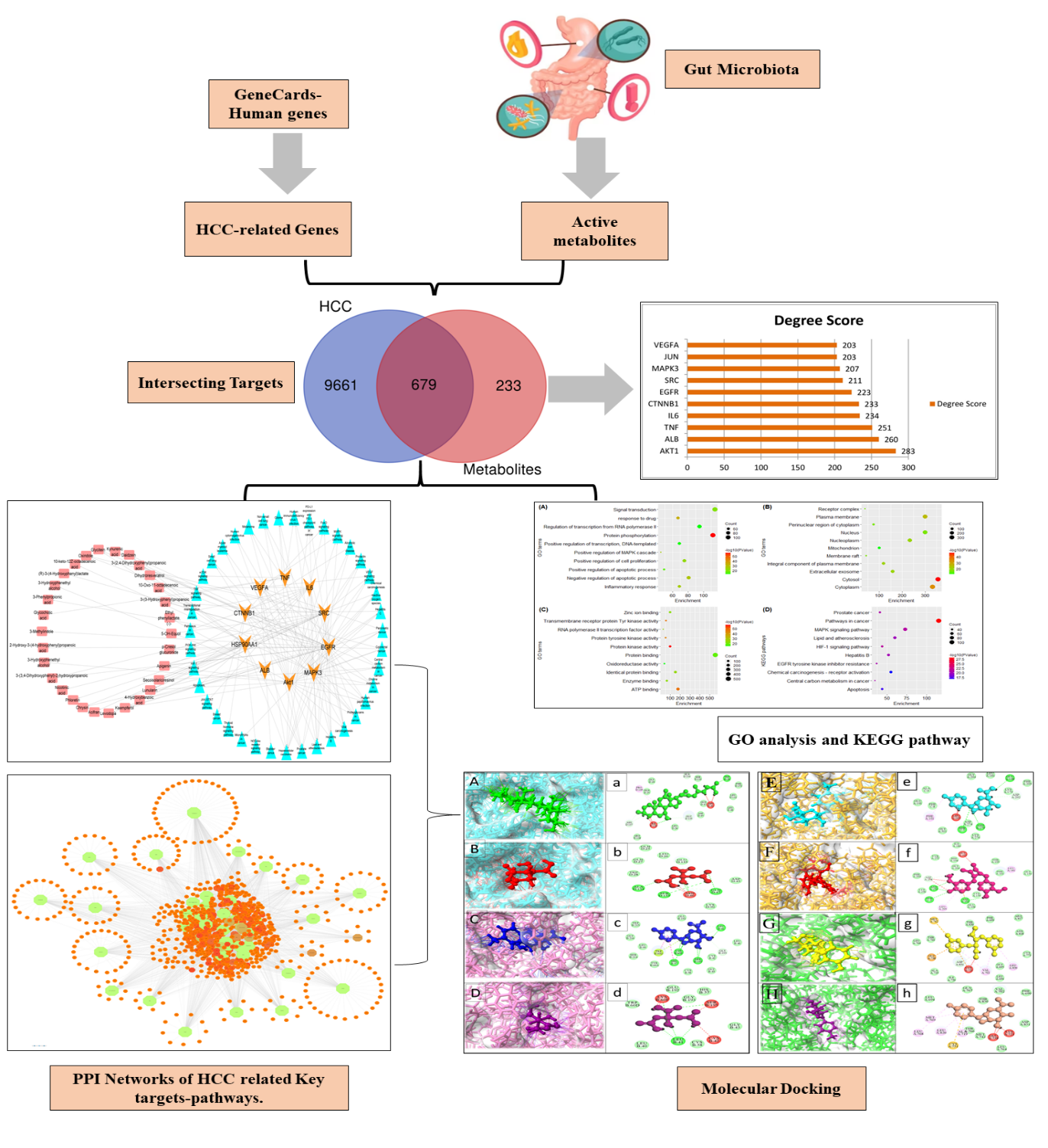
**

**Figure S1.** Graphical abstract illustrating the workflow used in current study

**Table S1: Selected Metabolites of Gut microbiota and their Physiochemical Properties.**

| **Metabolites** | **Mol. W**  **(g/mol)** | **Lipinski rule** | | | **Lipinski’s Violation** | **Bioavailability Score** | **Topological Surface area** | **Drug Likeness** |
| --- | --- | --- | --- | --- | --- | --- | --- | --- |
|  |  | **H-bonding Acceptor** | **H-bonding**  **Donor** | **MLogp** |  |  |  |  |
| Trimethylamine oxide | 75.11 | 1 | 0 | -1.66 | 0 | 0.55 | 29.43 Å² | -1.15 |
| D-Lactic acid | 90.08 | 3 | 2 | -0.85 | 0 | 0.85 | 57.53 Å² | -0.20 |
| 3-(3-Hydroxyphenyl) propanoic acid | 166.87 | 3 | 2 | 1.37 | 0 | 0.85 | 57.53 Å² | 0.29 |
| 3-Hydroxyphenethyl alcohol | 138.16 | 2 | 2 | 1.21 | 0 | 0.55 | 40.46 Å² | 0.29 |
| 5-OH-Equol | 258.27 | 4 | 3 | 1.52 | 0 | 0.55 | 69.52 Å² | 0.17 |
| 2-(4-Hydroxyphenyl) propionic acid | 166.17 | 3 | 2 | 1.37 | 0 | 0.55 | 57.53 Å² | 0.12 |
| 3-Hydroxybenzoic acid | 138.12 | 3 | 2 | 1.37 | 0 | 0.99 | 57.33 Å² | -0.60 |
| 4-Hydroxybenzoic acid | 138.12 | 3 | 2 | 1.37 | 0 | 0.85 | 57.33 Å² | -0.37 |
| Sedoheptulose | 210.18 | 7 | 6 | 3.36 | 0 | 0.85 | 138.45 Å² | -0.44 |
| Citric acid | 192.12 | 7 | 4 | -1.48 | 0 | 0.56 | 138.16 Å² | 0.52 |
| Tricarballylic acid | 176.12 | 6 | 3 | -0.68 | 0 | 0.55 | 111.90 Å² | -0.53 |
| Pipecolic acid | 129.16 | 3 | 2 | -2.21 | 0 | 0.56 | 49.33 Å² | -1.26 |
| Malic acid | 134.09 | 5 | 3 | -1.37 | 0 | 0.56 | 94.83 Å² | -0.50 |
| Tartaric acid | 150.09 | 6 | 4 | -2.18 | 0 | 0.56 | 115.06 Å² | 0.59 |
| Creatine | 131.13 | 3 | 3 | -0.96 | 0 | 0.55 | 90.41 Å² | -0.72 |
| 2-Imino-1-methylimidazolidin-4-one | 113.12 | 2 | 2 | -0.93 | 0 | 0.56 | 56.19 Å² | -0.51 |
| Glycocholic acid | 465.62 | 6 | 5 | 2.15 | 0 | 0.56 | 127.09 Å² | 0.29 |
| 3-Phenylpropionic acid | 150.17 | 2 | 1 | 1.98 | 0 | 0.85 | 37.30 Å² | -1.23 |
| Enterodiol | 302.36 | 4 | 4 | 2.2 | 0 | 0.55 | 80.92 Å² | -0.72 |
| (2R)-1-(3,4-dihydroxyphenyl)-3-(2,4,6-trihydroxyphenyl) -propan-2-ol | 292.28 | 6 | 6 | 0.64 | 0 | 0.55 | 121.38 Å² | 0.55 |
| Lactate | 89.07 | 3 | 1 | -0.85 | 0 | 0.85 | 60.36 Å² | -1.09 |
| Daidzein | 254.42 | 4 | 2 | 1.08 | 0 | 0.55 | 70.67 Å² | 0.29 |
| Levodopa | 197.19 | 5 | 4 | -2.26 | 0 | 0.55 | 103.78 Å² | 0.58 |
| L-Lactate | 89.07 | 3 | 1 | -0.85 | 0 | 0.85 | 60.36 Å² | -1.09 |
| Chrysin | 254.24 | 4 | 2 | 1.08 | 0 | 0.55 | 70.67 Å² | -0.21 |
| Apigenin | 274.24 | 5 | 3 | 0.52 | 0 | 0.55 | 90.90 Å² | 0.39 |
| Acetyl phosphate(2-) | 138.02 | 5 | 0 | -1.53 | 0 | 0.56 | 99.30 Å² | -1.34 |
| Oxindole | 133.15 | 1 | 1 | 1.13 | 0 | 0.55 | 29.10 Å² | -1.32 |
| 3-Methylindole | 131.17 | 0 | 1 | 1.87 | 0 | 0.55 | 15.79 Å² | -1.85 |
| Kynurenic acid | 189.17 | 4 | 2 | -0.35 | 0 | 0.85 | 70.42 Å² | 0.07 |
| Histidine | 155.15 | 4 | 3 | -3.74 | 0 | 0.55 | 92.00 Å² | -0.21 |
| 2-Hydroxy-3-(4-hydroxyphenyl)propanoic acid | 182.17 | 4 | 3 | 0.52 | 0 | 0.56 | 77.76 Å² | -0.14 |
| 3-(3,4-Dihydroxyphenyl)-2-hydroxypropanoic acid | 198.17 | 5 | 4 | -0.04 | 0 | 0.56 | 97.99 Å² | 0.57 |
| Nicotinic acid | 123.11 | 3 | 1 | -1.13 | 0 | 0.85 | 50.19 Å² | 0.30 |
| (R)-3-Hydroxybutyrate | 103.10 | 3 | 1 | -0.39 | 0 | 0.85 | 60.36 Å² | -1.51 |
| Acifran | 218.81 | 4 | 1 | 0.57 | 0 | 0.85 | 63.60 Å² | -0.23 |
| (S)-3-Hydroxybutyric acid | 104.10 | 3 | 2 | -0.39 | 0 | 0.85 | 57.53 Å² | -1.05 |
| Sodium 3-hydroxybutyrate | 126.09 | 3 | 1 | -0.39 | 0 | 0.55 | 60.36 Å² | -1.51 |
| Valerate | 101.12 | 2 | 0 | 0.89 | 0 | 0.85 | 40.13 Å² | -1.13 |
| Dihydroresveratrol | 230.26 | 3 | 3 | 2.34 | 0 | 0.55 | 60.69 Å² | -0.49 |
| Lunularin | 214.26 | 2 | 2 | 2.95 | 0 | 0.55 | 40.46 Å² | -0.33 |
| Colibactin | 770.88 | 11 | 4 | -2.54 | 2 | 0.17 | 274.59 Å² | 0.59 |
| beta-D-Fructofuranose | 180.16 | 6 | 5 | -2.74 | 0 | 0.55 | 110.38 Å² | -0.97 |
| Genistein | 270.24 | 5 | 3 | 0.52 | 0 | 0.55 | 90.90 Å² | 0.44 |
| Isovaleric acid | 102.13 | 2 | 1 | 0.89 | 0 | 0.85 | 37.30 Å² | -1.51 |
| Palmitic acid | 256.42 | 2 | 1 | 4.19 | 0 | 0.85 | 37.30 Å² | -0.54 |
| 10-Keto-12Z-octadecenoic acid | 296.44 | 3 | 1 | 3.59 | 0 | 0.85 | 54.37 Å² | -0.21 |
| Folic acid | 441.40 | 9 | 6 | -1.03 | 2 | 0.11 | 213.28 Å² | 1.18 |
| Quinic acid | 192.17 | 6 | 5 | -2.14 | 0 | 0.56 | 118.22 Å² | -1.06 |
| Oxalacetic acid | 132.07 | 5 | 2 | -1.47 | 0 | 0.56 | 91.67 Å² | -1.23 |
| Serotonin | 176.22 | 2 | 3 | 0.67 | 0 | 0.55 | 62.04 Å² | -0.98 |
| Glycerol | 92.09 | 3 | 3 | -1.51 | 0 | 0.55 | 60.69 Å² | -0.99 |
| Genipin | 226.23 | 5 | 2 | 0.12 | 0 | 0.56 | 75.99 Å² | -0.44 |
| 10-Oxo-11-octadecenoic acid | 296.44 | 3 | 1 | 3.59 | 0 | 0.85 | 54.37 Å² | -0.42 |
| Lacto-N-tetraose | 707.63 | 22 | 14 | -7.65 | 3 | 0.17 | 360.19 Å² | 0.15 |
| beta-D-Gal-(1->4)-beta-D-GlcNAc-(1->3)-beta-D-Gal-(1->4)-D-Glc | 707.63 | 21 | 14 | -8.05 | 3 | 0.17 | 356.70 Å² | 0.27 |
| p-Cresol glucuronide | 284.46 | 7 | 4 | -0.74 | 0 | 0.56 | 116.45 Å² | -1.15 |
| Secoisolariciresinol | 364.42 | 6 | 4 | 1.56 | 0 | 0.55 | 99.38 Å² | -0.40 |
| Phloretin | 274.27 | 5 | 4 | 1.1 | 0 | 0.55 | 97.99 Å² | 0.09 |
| Isobutyric acid | 88.11 | 2 | 1 | 0.49 | 0 | 0.85 | 37.30 Å² | -1.18 |
| (R)-3-(4-Hydroxyphenyl)lactate | 181.17 | 4 | 2 | 0.52 | 0 | 0.56 | 80.59 Å² | -0.39 |
| Ethyl phenyllactate, (-)- | 194.23 | 3 | 1 | 1.7 | 0 | 0.55 | 46.53 Å² | -0.74 |
| Kaempferol | 286.24 | 6 | 4 | -0.03 | 0 | 0.56 | 111.13 Å² | 0.50 |
| Glycitein | 284.26 | 5 | 2 | 0.77 | 0 | 0.55 | 79.90 Å² | 0.29 |
| Loganetin | 228.24 | 6 | 2 | 0.22 | 0 | 0.56 | 75.99 Å² | -0.44 |
| 1,3-Diphenylpropan-2-ol | 212.29 | 1 | 1 | 3.6 | 0 | 0.55 | 20.23 Å² | -0.90 |
| 2,4-Dioxopentanedioic acid | 160.08 | 6 | 2 | -1.96 | 0 | 0.56 | 108.74 Å² | -1.38 |
